# Serum Scavenging Capacity and Folliculogenesis Impact following Flaxseed Consumption in the First-Generation Mice Pups

**DOI:** 10.1155/2022/5342131

**Published:** 2022-05-30

**Authors:** Fahimeh Pourjafari, Tahereh Haghpanah, Maria Grazia Palmerini, Massood Ezzatabadipour

**Affiliations:** ^1^Anatomical Sciences Department, School of Medicine, Kerman University of Medical Sciences, Kerman, Iran; ^2^Department of life, Science and Environmental Sciences, University of L'Aquila, L'Aquila, Italy

## Abstract

Flaxseed is a source of antioxidants utilized for female infertility treatment in traditional medicine. This study investigated the effects of flax hydroalcoholic extract and flaxseeds during prenatal and postnatal (PND) periods on folliculogenesis and serum total antioxidant capacity (TAC). Pregnant NMRI mice received 500 and 1000 mg/kg of flax extract (LE) and the same doses of flaxseed (LS). Female pups received the same regimen for 56 days. The body, ovarian morphometry, follicle development, and TAC levels were evaluated. The ovarian weight significantly increased in the LE1000 group compared to the LS500 group. The LE500 group had a considerably lower number of primary and antral follicles compared to the CTL and LS1000 groups. The number of antral follicles significantly increased in the LE1000 group compared to the LS500 and LE500 groups. The number of preovulatory follicles was higher in the LE1000 group. A significant increase in the TAC levels was detected in the LS500, LS1000, and LE1000 groups. LE showed a dose-dependent protective effect on the folliculogenesis in F1, which is more evident with the dosage of 1000 mg/kg. This could be related to the strongest antioxidant property of LE1000, as shown by the highest levels of TAC.

## 1. Introduction

The ovary is a reservoir of follicles that, with the secretion of hormones influenced by the hypothalamic-pituitary axis, periodically induces follicular waves. Progression of small primordial follicles into large preovulatory follicles and, eventually, ovulation [1] can be affected by internal and external environmental factors, including free radicals [2]. During folliculogenesis, most of the follicles undergo atresia by apoptosis [3]. Antioxidants prevent follicular atresia by scavenging environmental free radicals, thereby improving folliculogenesis, maintaining effective ovulation [4], and women's health and fertility. Therefore, the use of antioxidant supplementation in assisted reproductive technologies (ARTs) is increasing [5].

Due to the expensive and challenging access to modern medicine, especially in some areas, herbal medicine is expanding [6]. *Linum usitatissimum*, known as flaxseed or linseed, is one of the traditional plants with a high concentration of biologically active components such as *α*-linolenic acid, lignans, unique proteins, and also potent antioxidants including phenolic acids and flavonoids [7]. The efficiency of flaxseed on human health is attributed to its high content of antioxidants. The effect of flaxseed on many body organs, including the reproductive system, has been demonstrated by many researchers. Following flaxseed consumption, a significant increase in the mouse body weight and size of the uterus and ovary as the promotion of bovine ovarian cycle, blood progesterone level, ovarian follicle and corpus luteum growth, and developmental competence of oocytes was reported [8]. Evidence suggests a dose-dependent effect of the flaxseed, where a 5% dose delayed puberty onset and increased the ovarian weight and a 10% dose led to early puberty onset, ovarian weight gain, serum estradiol elevation, and lengthened estrous cycles in female rats [9].

In contrast to the above data, we previously reported a detrimental effect of flaxseed on the murine ovarian follicular reserve, with increased serum estrogen levels and apoptotic gene expression [10].

Therefore, even if the effects of maternal diet during pregnancy and lactation on the proper development of the genital system and ovarian follicular reserve are well known [11], the present study aimed to evaluate the total antioxidant capacity of two doses of flaxseed (500 and 1000 mg/kg) on ovarian folliculogenesis in first-generation mice pups.

## 2. Materials and Methods

### 2.1. Preparation of Hydroalcoholic Extract of Flaxseed

Fresh flaxseeds (*Linum usitatissimum*) were purchased from an herbal medicine store in Kerman, Iran. The plant was identified by a botanist of the Herbarium of Pharmacognosy Department, School of Pharmacy, Kerman University of Medical Sciences, Kerman, Iran (voucher number: KF1628). Extraction was performed using a warm maceration method. Initially, a known amount (100 g) of the powder was prepared from the flaxseeds and then passed through a sieve. The powder was soaked with 80% ethanol for 72 h. Subsequently, the extract was concentrated on a rotary evaporator under vacuum conditions, dried in an oven at 40°C for 48 h, and stored at −20°C until use [12].

### 2.2. Animal Procedures

Forty female and twenty male NMRI mice (weighing about 25–30 *g*; 6–8 weeks old) were purchased from the animal house affiliated with Afzalipour School of Medicine, Kerman, Iran (approval number: IR.KMU.REC.1397.148). Animals were kept under controlled conditions (lighting, humidity, and temperature) with standard mice chow and water *ad libitum*.

In each cage, one male and two female mice were placed for mating overnight. The female mice were monitored daily for vaginal plaque formation. Pregnant mice were randomly divided into five groups: control (CTL) and four experimental groups. Each group included four mice. Animals in the control group received rodent food. The mice in the experimental groups received 500 mg/kg/day [13] or 1000 mg/kg/day [14] of flax hydroalcoholic extract (LE) or flaxseed (LS) (LE500, LS500, LE1000, and LS1000, respectively). Extracts and seeds were added to the diet food of pregnant mothers until the end of lactation and then continued for the female offspring mice until postnatal day 56 (PND56) (*n* = 7) [15].

### 2.3. Ovarian Histology

At the end of the experimental time point (PND 56), female mice pups were weighed and euthanized by cervical dislocation. The right ovaries of animals were excised, cleared of all the adhering fat, and weighed by using a digital scale. The large and small diameters were measured by using a digital caliper. The collected right ovaries were fixed in 10% formalin. The fixed tissue samples were dehydrated in ascending concentrations of alcohol and embedded in paraffin. Slices of tissue with 5 *µ*m thickness were prepared and stained with hematoxylin and eosin and then evaluated under a light microscope (Olympus IX51, Japan) [16].

### 2.4. Histological Classification of Ovarian Follicles, Follicle Count, and Stages

To count the number of follicles in the ovarian tissue, the prepared blocks were serially sliced, and for every ten serial sections, one slice was collected (8 sections for each sample, *n* = 7). The follicles were classified as (1) primordial if they contained an oocyte surrounded by one layer of flatted follicular cells; (2) primary if a relatively larger oocyte was surrounded by one layer of cuboidal follicular cells; (3) secondary if they contained two or three layers of cuboidal granulosa cells with no antral space; (4) preantral if they were enclosed in more than four layers of granulosa cells with one or more independent spaces; (5) antral if they contained multiple layers of cuboidal granulosa cells with a specified antral space; (6) preovulatory if an antral space divided the mural granulosa cells from the oocyte and its cumulus oophorus; (7) atretic if they contained fragmented oocyte, ruptured plasma membrane, and dropped granulosa cells into the antrum. Corpora lutea, forming large steroid-producing organs, were also counted [17].

### 2.5. Assessment of Serum Total Antioxidant Capacity (TAC) Level

The animals were anaesthetized with ketamine (5–10 mg/kg) and xylazine (5 mg/kg). Their blood samples were collected directly from the left ventricle of the heart and centrifuged at 2000–2500 rpm for 10 minutes. Serum samples were stored at −20°C until assay for the TAC level. The serum TAC level was measured by the ferric ion reducing antioxidant power (FRAP) method according to the TAC commercial kit (Cat no. NS-15012, Navand Salamat Company, Urmia, Iran) [18].

### 2.6. Statistical Analysis

The normality of the distribution of continuous data was tested using the Kolmogorov–Smirnov test. In the comparison of continuous variables with more than two independent groups, the ANOVA test was used when the normal distribution condition was met, with the Tukey test as a post hoc. The Kruskal–Wallis test was used for nonparametric variables. Data were expressed as mean ± SEM. A *P* value ≤0.05 was considered statistically significant.

## 3. Results

### 3.1. Body Weight, Ovarian Weight, and Ovarian Size

As shown in Table 1, the body weight and ovarian size of mice that were daily treated with flaxseed and flax extract at different doses (500 and 1000 mg/kg) did not show significant changes. However, when comparing the ovarian weights among the groups, an increase of this parameter was observed in the LE1000 group compared to other groups, although this reached a significant difference when compared to the LS500 group (4.8 ± 0.5 *vs.* 3.7 ± 0.67, respectively, *P* < 0.05) (Table 1).

### 3.2. Morphological and Morphometric Study of the Ovaries

**Figure 1:**
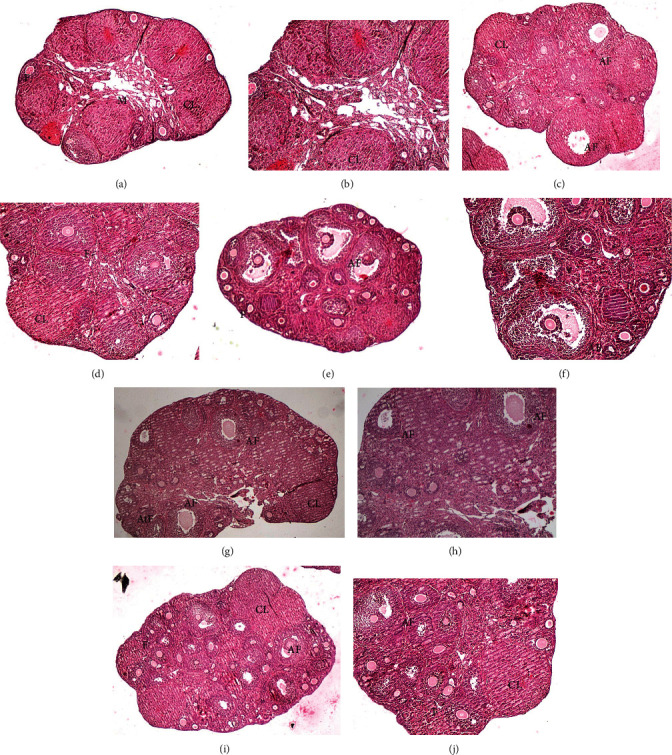
Photomicrographs of representative ovarian sections from the different experimental groups stained by hematoxylin and eosin. (a, b) Control group showing a spongy stroma in the medulla (M) and a cortex rich in small follicles, corpora lutea (CL), and also corpora hemorragica (*asterisk*) (40× and 100× magnifications). (c, d) The LS500 group with a dense cortex with numerous secondary and tertiary follicles and corpora lutea (CL) (40× and 100× magnifications); AF, antral follicles. (e, f) The LS1000 group showing numerous secondary and antral follicles, with visible cumulus-oocyte complexes inside the antral cavity (40× and 100× magnifications). (g, h) The LE500 group with growing and antral follicles (AF), corpora lutea (CL), and numerous atretic follicles (AtF) (40× and 100× magnifications). (i, j) LE1000 with numerous growing and antral follicles (AF) and corpora lutea (CL) (40× and 100× magnifications).

In controls, the ovarian cortex was densely populated by numerous primary, secondary, and tertiary (preantral) follicles interspersed among numerous big corpora lutea and corpora hemorrhagica (as evidenced by cells intensely stained in red). Small atretic follicles were also seen. The medulla had a highly vascularized and spongy stroma (Figures 1(a) and 1(b)). Ovaries from the experimental groups (LS500, LS1000, LE500, and LE1000) are rich in follicles at different developmental stages and corpora lutea. Numerous antral follicles with visible cumulus-oocyte complex are detectable (Figures 1(c)–1(j)). The medulla had a more compact aspect, probably connecting the cutting sections. Small atretic follicles were occasionally detected in all groups.

**Figure 2:**
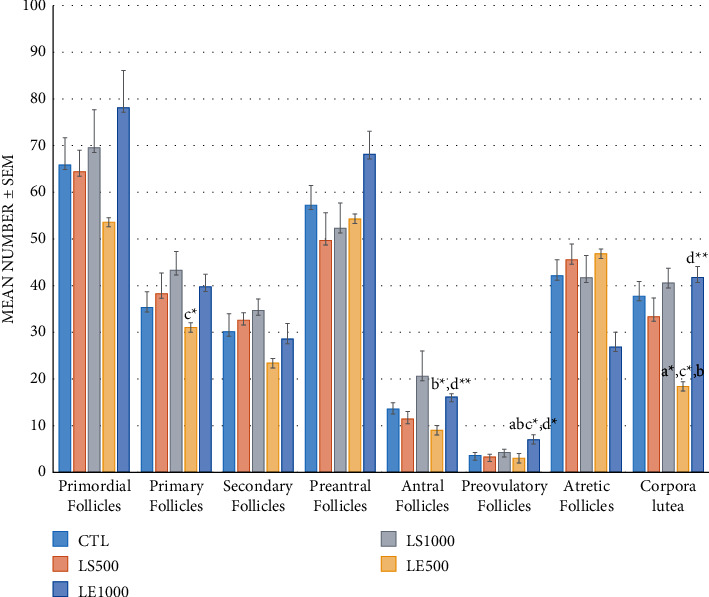
Effect of *in vivo* administration of hydroalcoholic extract and flaxseed on the number of different follicles including primordial, primary, secondary, preantral, antral, preovulatory, and atretic follicles. The values are represented as mean ± SEM (*n* = 7/group). ^a^A significant difference in comparison with the CTL group. ^b^A significant difference in comparison with the LS500 group. ^c^A significant difference in comparison with the LS1000 group. ^d^A significant difference in comparison with the LE500 group. The statistical values ^*∗*^*P* < 0.05, ^*∗∗*^*P* < 0.01, and ^*∗∗∗*^*P* < 0.001 represent a significant difference among the groups.

The morphometric analysis evidenced that the number of primordial, secondary, preantral, and atretic follicles in the offspring ovaries did not show significant differences among all the experimental groups (Figure 2), even if the lowest number of primordial follicles was found in the LE500 group (53.57 ± 12.87) and the highest in the LE1000 group (78.14 ± 7.91) (*P* > 0.05) (Figure 2(a)). The number of atretic follicles was contrariwise in these two groups (46.85 ± 5.38 and 26.85 ± 3.15 in the LE500 and LE1000 groups, respectively) (Figure 2(d)). Treatment with the flax hydroalcoholic extract at a dose of 500 mg/kg (LE500) (31 ± 3.06) caused a significant reduction in the number of primary follicles in the ovarian parenchyma compared to the LS1000 group (43.28 ± 4.01) (*P* < 0.05) (Figure 2(a)).

Furthermore, a marked decrease in the number of antral follicles was found in the ovaries of LE500 (9 ± 1.66) treated mice compared to the CTL group (13.57 ± 1.37) (*P* < 0.05) (Figure 2(c)). The number of antral follicles significantly increased in the LE1000 group (16.14 ± 0.63) compared to the LS500 (11.42 ± 1.57) and LE500 groups (LE1000 *vs*. LS500; LE1000 *vs*. LE500; *P* < 0.05 and *P* < 0.001, respectively) (Figure 2(c)). The number of preovulatory follicles significantly increased in LE1000 (7 ± 1.06) ovaries in comparison with the other groups (3.57 ± 0.68, 3.28 ± 0.64, 4.28 ± 0.68, and 3 ± 0.53 in the CTL, LS500, LS1000, and LE500 groups, respectively) (CTL, LS500, and LS1000; *P* < 0.05, and also versus LE500; *P* < 0.01). No differences were found in the number of corpora lutea among CTL, LS500, LS1000, and LE1000 groups. Only in the LE500 group (18.42 ± 3.65), a significant decrease with respect to CTL, LS1000, LE1000, and LS500 was observed (37.71 ± 3.16, 40.57 ± 3.14, 41.71 ± 2.28, and 33.28 ± 4.05, respectively) (*P* < 0.01 versus CTL, LS1000, and LE1000 and *P* < 0.05 versus LS500) (Figure 2(d)).

### 3.3. Total Antioxidant Capacity (TAC) Assay

**Figure 3:**
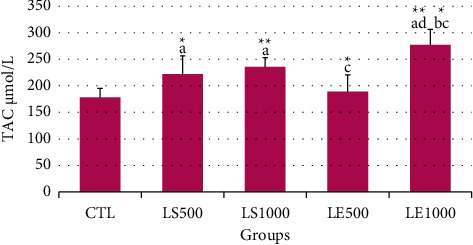
Effect of prenatal and postnatal exposure to flax hydroalcoholic extract or flaxseed on the total antioxidant capacity (TAC) level in the serum of female offspring mice. ^a^A significant difference compared to the CTL group. ^b^A significant difference compared to the LS500 group. ^c^A significant difference compared to the LS1000 group. ^d^A significant difference compared to the LE500 group. The values are represented as mean ± SEM. ^*∗*^ and ^*∗∗*^indicate *P* < 0.05 and *P* < 0.01, respectively.

Flaxseed treatment at the two different tested doses elevated the TAC level with respect to the CTL group (177.67 ± 16.83), with a great increase at the highest dose (221.77 ± 33.72 and 234.77 ± 16.71 in the LS500 and LS1000, respectively) (CTL *vs.* LS500, *P* < 0.05; CTL *vs.* LS1000, *P* < 0.01). Statistical analysis showed a significantly higher TAC level in the LE1000 group (275.8 ± 29.7) than in the other groups (177.67 ± 16.83, 187.89 ± 31.71, 221.77 ± 33.72, and 234.77 ± 16.71 in the CTL, LE500, LS500, and LS1000 groups, respectively) (LE1000 *vs.* CTL, LE1000 *vs.* LE500, *P* < 0.01; LE1000 *vs.* LS1000, LE1000 *vs*. LS500; *P* < 0.05). However, the lowest level of TAC was observed in the LE500 group (187.89 ± 31.71 and 234.77 ± 16.71 in the LE500 and LS1000 groups, respectively) (*P* < 0.05 versus the LS1000 group) (Figure 3).

## 4. Discussion

Among herbs, those with antioxidant properties have attracted much attention worldwide. Diet is one way to get antioxidants. Dietary antioxidants can fight free radicals [19]. Recently, flaxseed has been shown to have therapeutic potential with anticancer, antiviral, bactericidal, anti-inflammatory, and antioxidant effects [20–22]. This medicinal herb contains numerous amounts of phenolic compounds with high antioxidant activities making it a potential source for treating degenerative diseases [23]. Flaxseed antioxidant effects on various organs such as the liver and kidney in rats exposed to thioacetamide have been reported. Administration of flaxseed could induce improvement in the liver function test, antioxidant status, and lipid profile. Also, this herb could reduce kidney necrosis, degeneration, and inflammatory cell infiltration [19, 24]. *In vitro*, antioxidant activities of flaxseed extracted with different methods, including methanolic and butanolic extracts, in addition to those acquired using petroleum ether, benzene, and ethyl acetate, have been evaluated. The butanolic extract of flaxseed showed the highest levels of DPPH (*α*,*α*-diphenyl-*β*-picryl hydroxyl) radical scavenging activity. In contrast, its petroleum ether and ethyl acetate extracts exhibited a significantly increased level of hydrogen peroxide scavenging activity. An increase in the ferric reducing antioxidant power (FRAP) was observed in its methanolic extract [25]. Since different extraction methods exhibited different scavenging activity levels, this study aimed to assess which, from the seed itself or the hydroalcoholic extract, has a higher ferric reducing antioxidant power (FRAP). The results of the study showed a significant difference between them based on FRAP; a daily intake of hydroalcoholic extract of flaxseed during intrauterine life, lactating, and then until puberty caused a significantly higher level of FRAP than the consumption of flaxseed did. Moreover, as mentioned earlier, this effect was dose-dependent, with the 1000 mg/kg dose showing the greatest effect compared to the 500 mg/kg dose.

Previous reports have determined that the hydroalcoholic extract of flaxseed (200 mg/kg for 7 weeks) could improve the side effects of PCOS on the hormonal profile and histomorphometric features of the ovaries [26]. Others evidenced that when the bovine pregnant basal diet is supplemented with flaxseed (8% of the basal diet), there is an amelioration of the ovarian cycle, the number of ovarian follicles, and corpora lutea, as well as the oocyte developmental competence; differently, a higher dose of flaxseed (>8%) induced negative effects [27–29].

In line with the data available, we observed different effects of the extract and seed based on the exposure dose. According to histological data and TAC results, the dose of 1000 mg/kg of flax was more effective on folliculogenesis as compared to 500 mg/kg of flax in both the two forms of the extract and seed, as evidenced by a higher number of antral and preovulatory follicles. Among the tested treatments, animals receiving 1000 mg/kg of hydroalcoholic extract of flaxseed showed the highest increase in the number of ovarian follicles in comparison with the other treatment groups. Moreover, an increase in the number of corpora lutea, with respect to the LE500 group, was also observed, thus evidencing an improvement in ovarian physiology. Among the treatments, the lower dose (500 mg/kg) did not show significant differences with respect to the control and other groups, except for a significant reduction of primary follicles and corpora lutea in the form of extract (LE500). In our recent study, it was observed that the dietary intake of 500 mg/kg of hydroalcoholic extract of flaxseed may affect ovarian reserve in the first-generation mouse pups by decreasing the number of primordial and growing follicles in addition to decreasing body and ovary weight. We also evaluated the expression of the anti-Müllerian (AMH) hormone as an ovarian reserve marker in the ovaries of adult female pups. Our data showed that flaxseed at a dosage of 500 mg/kg could decrease the AMH expression [30]. These data were probably explained by the antiestrogenic property of the hydroalcoholic extract of flaxseed at the 500 mg/kg dosage [10].

In this study, we also reported that flaxseed at doses of 500 and 1000 mg/kg and also the hydroalcoholic extract of flaxseed only at 1000 mg/kg dosage can increase body and ovarian weight. This effect was not observed at 500 mg/kg of hydroalcoholic extract of flaxseed. In other studies, it has been suggested that body weight gain following flax intake is probably due to the estrogenic properties of this plant. Also, an increase in the number of growing follicles induced by flaxseed in different treated groups, except for the 500 mg/kg hydroalcoholic extract group, could lead to an increase in ovarian weight. This result is in agreement with previous observations that have reported that dietary flaxseed (10%) could cause a lower birth weight of mouse pups and female offspring with shorter anogenital distance, greater relative uterine, and ovarian weights, as well as earlier age, lower body weight at puberty, and a lengthened estrous cycle [9]. Another study determined that 5% dietary flaxseed led to a delay in the onset of puberty in females, whereas 10% dietary flaxseed could cause an earlier puberty onset, higher relative ovarian weight, and also a lengthened estrous cycle [9].

The novel data presented here about the improvement of folliculogenesis and the total antioxidant capacity (TAC) connected to the consumption of different forms (extract and seed) and diverse doses (500 and 1000 mg/kg) of flaxseed are the basis for future studies to confirm the potential applications of flaxseed supplementation to enhance the reproductive performance in physiological and pathological conditions.

## 5. Conclusion

The protective effects of consumption and antioxidant properties of flaxseed on folliculogenesis and fertility of women may depend on dosage and the form of consumption.

## Figures and Tables

**Table 1:** Effect of hydroalcoholic extract and flaxseed on the body weight, ovarian weight, and ovarian diameters of female offspring mice.

Groups	Variables			
	Mice weight (*g*)	Ovarian weight (mg)	Large diameter (mm)	Small diameter (mm)
CTL	21.75 ± 0.66	3.2 ± 0.68	2.37 ± 0.15	1.79 ± 0.17
LS500	21.92 ± 0.86	3.1 ± 0.34	2.03 ± 0.22	1.18 ± 0.10
LS1000	22.27 ± 0.57	3.7 ± 0.67	2.22 ± 0.16	1.38 ± 0.13
LE500	20.98 ± 0.52	3 ± 0.72	1.72 ± 0.24	1.47 ± 0.14
LE1000	23.18 ± 0.7	4.8 ± 0.5^b^^*∗*^	2.15 ± 0.2	1.63 ± 0.11

The values are expressed as mean ± SEM. The values are comparable in the same column, ^b^Significant difference versus LS500 group; ^*∗*^*p* < 0.05.

## Data Availability

The research data used to support the findings of this study are available. Request for access to these data should be made to Ezzatabadipour Massood (Corresponding author, m_eatabadi@kmu.ac.ir, ezzatabadim@gmail.com).
